# Antibody and aptamer-based therapies for osteoarthritis: Application of antibodies and promise of aptamers

**DOI:** 10.1016/j.omtn.2025.102552

**Published:** 2025-05-05

**Authors:** Huaizhi Chen, Zijian Ye, Jasmijn V. Korpershoek, Laura B. Creemers, Harrie Weinans, Jaqueline Lourdes Rios

**Affiliations:** 1 Department of Orthopedic Surgery, University Medical Center Utrecht, 3584 CX Utrecht, the Netherlands; 2 Department of Orthopedic Surgery, Mayo Clinic, Rochester, MN 55905, USA; 3 Department of Biomechanical Engineering, Faculty of Mechanical Engineering, Delft University of Technology (TU Delft), 2628 BX Delft, the Netherlands

**Keywords:** MT: Oligonucleotides: Therapies and Applications, targeted therapy, osteoarthritis, monoclonal antibodies, nucleic acid aptamers

## Abstract

Osteoarthritis (OA) is a common degenerative inflammatory joint disease with progressive loss of articular cartilage that undermines patients’ quality of life. There are no regulatory-approved, disease-modifying OA medications, despite a great deal of studies done to elucidate OA pathogenesis. Until now, OA pharmacological treatment focused mainly on generalized inhibition of inflammation and pain. Currently, monoclonal antibodies and nucleic-acid aptamers emerge as targeted therapies offering potential alternatives by addressing the complex challenges posed by OA, such as specifically reducing inflammation and pain in the joint targeting specific molecular key players, instead of a systemic and generalized approach like with non-steroidal anti-inflammatory drugs. Aptamers’ properties, including structure versatility, reduced immunogenicity, and flexible administration methods, position them as high-potential candidates for OA treatment. This review summarizes results from clinical trials applying monoclonal antibodies to treat OA, preclinical research, and the development of aptamers as a new generation of targeting agents. Meanwhile, it provides a comprehensive comparison of the characteristics, advantages, and limitations of aptamers versus monoclonal antibodies. Notably, the promising applications of aptamers, demonstrated in other inflammatory and degenerative conditions, underscore their potential for OA therapy. We anticipate that the application of aptamer could offer a new way of OA pharmacological intervention.

## Graphical abstract

**Figure:**
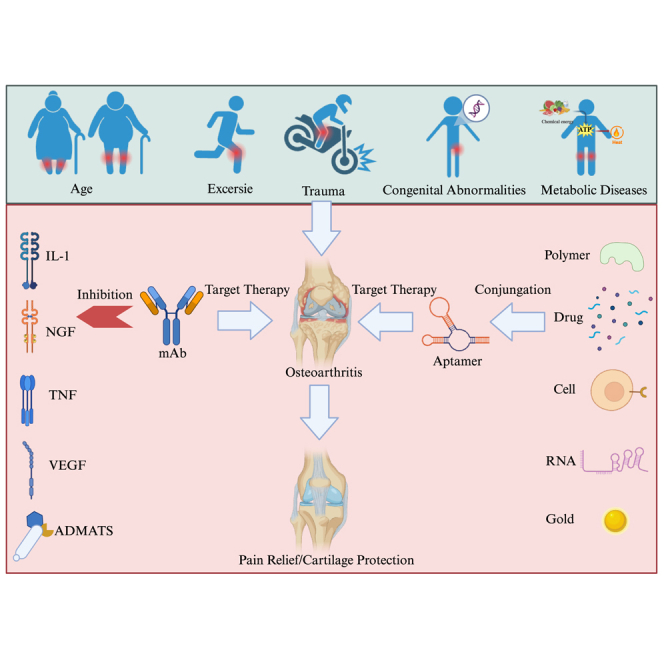

## Teaser

Despite decades of clinical trials, mAbs have yet to achieve clinical success in osteoarthritis (OA) treatment due to limited efficacy and adverse events. Based on a review of mAb clinical trials and a comparison of mAb and aptamers, we propose aptamers hold significant promise for OA treatment.

## Introduction

Osteoarthritis (OA) is the most prevalent chronic joint disease, characterized by gradual progressive damage of the whole joint, including synovial hyperplasia, articular cartilage loss, and osteophyte formation.^1^^,^^2^^,^^3^ Due to pain, decreased mobility, and diminished joint function, OA significantly lowers the life quality of patients and poses financial pressure on society.^4^^,^^5^^,^^6^^,^^7^^,^^8^ Thus, the development of efficacious OA therapies is increasingly imperative.

There are three common approaches for OA treatment: pharmacological, non-pharmacological (such as physical therapy, moderate exercise, and assisted joint protection), and surgical therapies. Treatment for patients with OA often combines pharmacological and non-pharmacological approaches.^9^^,^^10^ However, in end-stage OA, surgical treatment, such as partial/total joint replacement, is often the only available therapy.^11^ Despite increasing insights into OA pathogenesis and the identification of potential targets, no treatment has yet been developed that selectively and effectively addresses these targets. A cure or disease-modifying therapy remains elusive—arguably the most significant unresolved challenge in the field. Although expanding knowledge of the signaling pathways involved in cartilage degradation has opened up opportunities for targeted therapies, the precise mechanisms driving the initiation and progression of OA are still not well understood. Decades of research into the cellular and molecular processes of OA have yet to yield a definitive breakthrough.^12^^,^^13^ Various cellular receptors—such as wingless-related integration site (Wnt), tumor necrosis factor (TNF), transforming growth factor β (TGF-β), fibroblast growth factor (FGF), and nerve growth factor (NGF)—along with key signaling pathways and regulators like AMP-activated protein kinase, mechanistic target of rapamycin/mammalian target of rapamycin (mTOR), bone morphogenetic protein, hypoxia-inducible factors, nuclear factor kappa-light-chain-enhancer of activated B cells, and interleukin-1 (IL-1)—may be involved in the pathogenesis of OA.^14^^,^^15^^,^^16^^,^^17^ Consequently, most targeted therapies for OA, particularly those based on recombinant proteins such as antibodies, are being developed to modulate these pathways.

**Figure 1:**
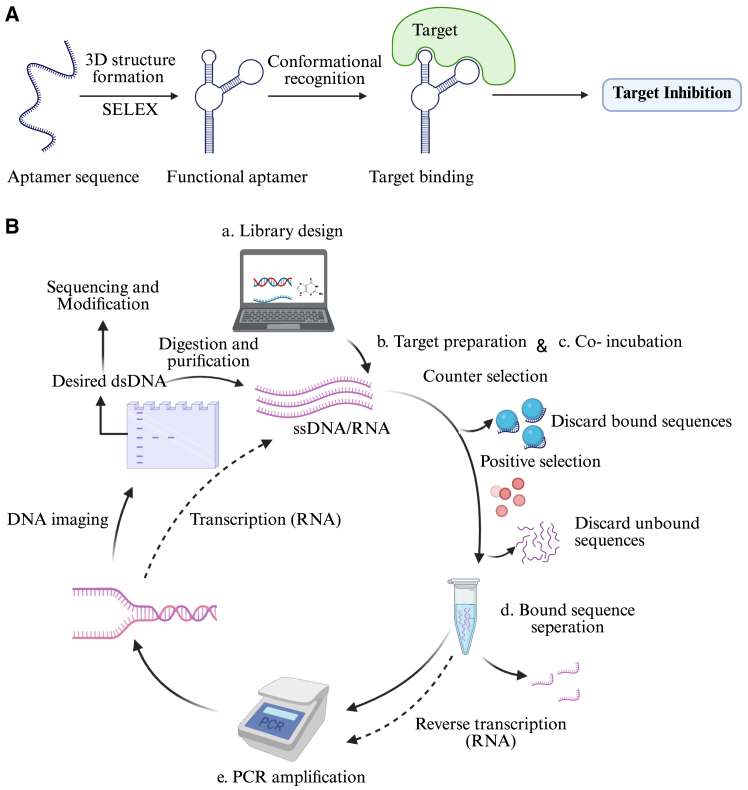
Schematic overview of aptamer selection via SELEX and target binding (A) Binding of aptamer to its target through conformational recognition. Through the SELEX process, an aptamer forms a unique three-dimensional (3D) structure that enables specific binding to its target, functioning as a highly selective inhibitor. (B) The essential steps of a standard SELEX technique. SELEX, systematic evolution of ligands by exponential enrichment; PCR, polymerase chain reaction; ssDNA, single-stranded DNA; dsDNA, double-stranded DNA; RNA, ribonucleic acid; DNA, deoxyribonucleic acid.

Other agents that specifically bind to targets include aptamers.^18^^,^^19^ Aptamers are synthetic short single-stranded nucleic acid sequences (ssDNA/RNA) with 40–100 nucleotides in length, which can bind to a broad spectrum of molecules with strong affinity and specificity, including proteins, peptides, nucleotides, antibiotics, toxins, and even small molecules^20^^,^^21^ (see Figure 1A). Aptamers are typically generated using an iterative, highly specific technique known as systematic evolution of ligands by exponential enrichment (SELEX) (see Figure 1B), which enables the identification of aptamers that selectively bind to their target molecules.^22^^,^^23^ The SELEX technique was published 34 years ago by two independent research groups,^18^^,^^19^ and a lot of improvements, such as capillary electrophoresis-SELEX, magnetic bead-based SELEX, and crossover SELEX, have been made since then to shorten the SELEX duration and make the screening process more cost-effective. In short, each SELEX round includes three main stages. Stage I—an aptamer library composed of a large number of random nucleic acid sequences (ssDNA or RNA), usually containing 10^9^–10^15^ different sequences,^24^ is incubated with the target molecules. Stage II—the unbound aptamers are discarded, after which the bound aptamers are recovered. Stage III—bound aptamers are eluted and amplified by PCR, and a new (enriched) aptamer library is created.^25^^,^^26^ The amplified nucleic acid is then used for the next round of screening to gradually enrich aptamers with high affinity and specificity. The entire SELEX cycle is then repeated, resulting in a new (enriched) library each round. As the number of rounds is increased, an increase in binding affinity of the aptamer candidates is observed, and about 8–15 rounds are needed to reach a pool of aptamers with strong binding.^27^^,^^28^ As a result, aptamers have been widely investigated as treatments, drug carriers, and diagnostic tools, and they have gained interest in the fields of functional genomics and bio-sensing.^29^^,^^30^^,^^31^^,^^32^^,^^33^

Here, we review the advantages and disadvantages of monoclonal antibodies (mAbs) and aptamers as emerging targeted treatment strategies for OA. We begin by examining recent advancements in mAb therapy for OA, including both past and ongoing clinical trials, and then summarize aptamer studies from various disease areas that could potentially be adapted for OA treatment. Finally, we provide a detailed comparison of mAbs and aptamers regarding their future applications in OA therapy, highlighting their respective clinical potentials. For a comprehensive overview of alternative molecular treatments for OA, see the review manuscript titled “Osteoarthritis: pathogenic signaling pathways and therapeutic targets” by Yao et al.^13^

## Selection of literature

The PubMed database (www.ncbi.nlm.nih.gov/pubmed) and clinical trial database of NIH (National Library of Medicine) (https://clinicaltrials.gov/) were used to select papers for inclusion in this narrative review. The following search key terms were employed for this purpose: conditions (osteoarthritis, arthritis, or OA), therapeutic agents (aptamer, antibody, or nucleic acid aptamers), and targets (IL-1, NGF, TNF, ADAMTS-5, or VEGF). To be included in this review, studies were required to be full-text articles published in English and contained at least one of the search terms in their title or abstract. Studies based on secondary analyses of previously published data from other articles or repositories were excluded. Literature reviews were not part of the data extraction process but were retained for contextual analysis and discussion.

According to our search, as of 2024, there are approximately 60 clinical trials worldwide focused on antibody-based therapies for OA. Among these, around 30 trials are currently ongoing, while approximately 15 trials have been terminated prematurely. This review highlighted an analysis of around 30 typical clinical trials investigating various mAb targets, including studies that are currently ongoing, completed, or terminated prematurely.

## Monoclonal antibodies for OA treatment

**Figure 2:**
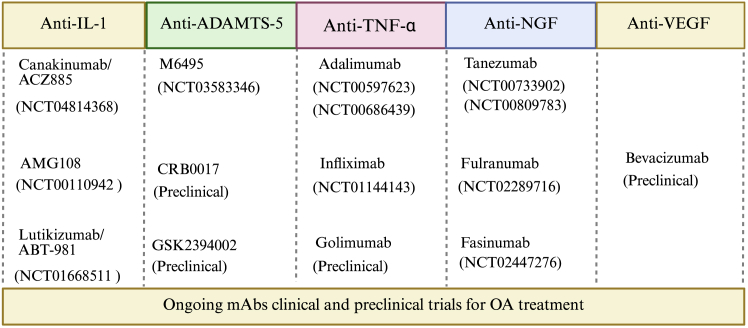
Clinical trials summary of monoclonal antibodies for OA treatment IL-1, interleukin-1; TNF, tumor necrosis factor; VEGF, vascular endothelial growth factor; NGF, nerve growth factor; ADAMTS-5, a disintegrin and metalloproteinase with thrombospondin motifs 5.

mAbs, produced exogenously to mimic the function of their endogenous counterparts, are designed to neutralize harmful molecules, which is a part of pathophysiological processes. Pharmaceutical companies and research institutions have actively collaborated to advance mAbs development for OA treatment. These antibodies have been investigated for their ability to target specific molecules implicated in OA pathogenesis, such as IL-1, a disintegrin and metalloproteinase with thrombospondin motifs 5 (ADAMTS-5), NGF, TNF, and vascular endothelial growth factor (VEGF) (see Figure 2).^34^ Several clinical trials have assessed the safety, efficacy, and potential benefits of mAbs in mitigating symptoms and modifying disease progression.^35^^,^^36^^,^^37^^,^^38^ Here, we provide an overview of recent clinical trials and related preclinical research with mAbs for OA treatment.

## Anti-IL-1 mAbs

Interleukin-1α (IL-1α) and interleukin-1β (IL-1β) are frequently detected in the joints of OA patients and are considered key orchestrators of the inflammatory response.^39^ Their levels are often elevated in OA synovial fluid, underscoring their significant role in the disease’s development and progression.^40^ In addition to inhibiting the synthesis of collagen and proteoglycans,^41^ IL-1 accelerates the production of nitric oxide and free radicals^42^ and induces the expression of extracellular matrix degrading proteases. As an inflammatory cytokine, IL-1 is also implicated in the activation of nociceptors in neuronal cells.^43^ Thus, targeting IL-1 with mAbs in OA may be a potentially useful strategy for both pain relief and slowing the disease’s development (see Table 1 for a summary of anti-IL-1 mAbs trials for OA treatment).

**Table 1:** Anti-IL-1 and anti-ADAMTS-5 mAbs for OA treatment

Generic name	Identifier (sponsor)	Mechanisms	Study phase	Outcome	Main findings	Study design	Last update	Reference
Canakinumab, ACZ885	NCT04814368 (Novartis)	anti-IL-1	phase 2 (recruiting)	not available	not available	intra-articularly injected canakinumab and/or LNA043 in knee OA patients	11-2023	–
AMG 108	NCT00110942 (Novartis)	–	phase 2 (terminated)	safety, uncertain efficacy	no statistical difference between placebo and AMG 108 groups	knee OA patients were given different dosage of placebo or AMG 108 subcutaneously once every 4 weeks for 12 weeks	06-2008	Cohen et al.^44^
Lutikizumab (ABT-981)	NCT01668511 (Novartis)	–	phase 1 (completed)	safety, efficacy	anti-inflammatory response (lower CRPs), injection site erythema (ABT-981 vs. placebo)	three groups got ABT-981 (0.3, 1, or 3 mg/kg) or placebo every other week, while one cohort got ABT-981 (3 mg/kg) or placebo each four weeks by SC injections in knee OA	11-2017	Wang et al.^45^
M6495	NCT03583346 (Merck)	anti-ADAMTS-5	phase 1 (completed)	not available	not available	subcutaneous injections of M6495 in participants with knee OA	01-2020	–
CRB0017	–		preclinical	efficacy	CRB0017 decreased disease progression by delaying cartilage breakdown compared to vehicle-treated mice	treated spontaneous OA male mice intra-articularly in each knee with CRB0017 for three months	11-2013	Wang et al.^46^
GSK2394002	–		preclinical	not available	ADAMTS-5 is the primary aggrecanase engaged in the breakdown of cartilage	employed *in vitro*, *ex vivo*, and *in vivo* systems to evaluate aggrecanase inhibition, and modulation of disease-related endpoints, respectively, by co-incubation or intra-articular injection	09-2023	Larkin et al.^47^

IL-1, interleukin-1; ADAMTS-5, a disintegrin and metalloproteinase with thrombospondin motifs 5; CRPs, C-reactive protein; SC, subcutaneous; OA, osteoarthritis.

Canakinumab (ACZ885) is a human immunoglobulin G kappa light chain (IgGκ) mAb that blocks IL-1β receptors.^36^ A clinical trial (NCT04814368) is currently underway to assess the efficacy, tolerability, and safety of intra-articular canakinumab in patients with knee OA. This phase 2 study is actively recruiting participants, and no prior results have been published. However, canakinumab was generally well tolerated by patients with no significant adverse events in clinical studies for treatment of cryopyrin-associated periodic syndromes, Still’s disease, and gouty arthritis.^36^^,^^48^^,^^49^ Canakinumab was superior to triamcinolone in terms of its ability to reduce the pain measured on the visual analogue scale score, serum amyloid A, and high sensitivity C-reactive protein (CRP) in patients with acute gouty arthritis, as demonstrated in a meta-analysis.^48^

Novartis has developed two anti-IL-1 mAbs, namely AMG 108 and lutikizumab (ABT-981). AMG 108, a fully humanized mAb of the immunoglobulin G2 subclass, specifically targets the IL-1 receptor type 1, effectively inhibiting the biological activities of both IL-1α and IL-1β isoforms.^44^ A randomized, double-blind study (NCT00110942) evaluated the therapeutic efficacy and safety of subcutaneous AMG 108 injections in patients with knee OA. In this trial, patients receiving AMG 108 did not exhibit significant pain relief, leading to the discontinuation of the AMG 108 development program due to insufficient evidence of clinical efficacy.^44^

ABT-981, which is also called lutikizumab, is an immunoglobulin G1 kappa light chain (IgG1/κ) subtype dual variable domain immunoglobulin (DVD-Ig) that neutralizes IL-1α and IL-1β.^46^^,^^50^ In a randomized phase 1 study (NCT01668511), the safety, tolerability, and pharmacodynamics of ABT-981 were evaluated in patients with knee OA. Subcutaneous administration of ABT-981 elicited an anti-inflammatory response by significantly reducing absolute neutrophil counts, type I collagen, CRP, and levels of both IL-1α and IL-1β. However, this treatment was associated with a higher incidence of injection site redness compared to placebo.^45^ Two phase 2 clinical studies (NCT02087904 and NCT02384538) aimed to assess the safety and therapeutic effectiveness of ABT-981 in knee OA and treating erosive hand OA (interphalangeal joints), respectively. In trial NCT02087904, IL-1 inhibition was generally ineffective as a pain-relieving and anti-inflammatory treatment for most patients with knee OA, which indicates that a higher dose might be needed to achieve sufficient IL-1 blocking and synovitis amelioration.^37^ Also, a clinical trial aiming to treat erosive hand OA by injecting ABT-981 subcutaneously failed to improve pain or imaging parameters indicative of structural effects when compared to placebo.^51^ In conclusion, together with published results from studies of other IL-1 inhibitors, the limited amelioration in the Western Ontario and McMaster Universities Arthritis Index (WOMAC) pain score and the lack of synovitis improvement may indicate that most individuals with knee OA and synovitis do not benefit from IL-1 inhibition as an analgesic or anti-inflammatory medication.^52^^,^^53^

## Anti-ADAMTS-5 mAbs

Cartilage degradation is a hallmark of OA, primarily resulting from the breakdown of extracellular matrix components such as aggrecan and collagens. Aggrecanases, which belong to the ADAMTS family of enzymes present in chondrocytes and synovial fibroblasts, play a key role in this process.^54^ In human joints, two aggrecanases have been identified: ADAMTS-4 (aggrecanase-1) and ADAMTS-5 (aggrecanase-2).^55^^,^^56^^,^^57^ Both enzymes share a similar domain structure that includes a pro-domain, a catalytic metalloproteinase domain, a disintegrin-like (Dis) domain, a cysteine-rich domain, and a spacer (Sp) domain, with ADAMTS-5 containing an additional thrombospondin (TS) domain following the Sp domain. ADAMTS-4 exhibits broader tissue distribution and higher expression levels in various adult tissues—particularly in the lungs, heart, and brain—while ADAMTS-5 shows a more restricted expression pattern, with substantial transcript levels predominantly in placental tissue.^58^ Emerging evidence from preclinical studies has demonstrated distinct roles of ADAMTS proteases in cartilage degeneration across species. While ADAMTS-5 has been identified as the predominant aggrecans responsible for cartilage degradation in murine models,^59^ accumulating evidence suggests that ADAMTS-4 may play a more significant role in the pathogenesis of cartilage degeneration in higher mammals, including humans.^60^^,^^61^^,^^62^ Studies indicate that inhibiting both ADAMTS-4 and ADAMTS-5 may be a good strategy to obtain optimum therapeutic effectiveness in treating OA.^58^^,^^61^ In line with this approach, the development of mAbs targeting these proteases is advancing rapidly, with two ADAMTS-5-specific mAbs (CRB0017 and GSK2394002) currently in preclinical evaluation and one candidate (NCT03583346) progressing to clinical trials (see Table 1 for a summary of anti-ADAMTS-5 mAbs trials for OA treatment).

CRB0017 is highly selective for its antigen and exhibits exceptional affinity in the low nanomolar range. In male mice, intra-articular administration of CRB0017 twice over three months delayed cartilage degradation in a dose-dependent manner, thereby altering the progression of OA.^63^ Despite these promising preclinical results, CRB0017 has not yet progressed to clinical trials. Similarly, GSK2394002—a mAb specifically targeting ADAMTS-5—is currently undergoing preclinical evaluation.^64^ In murine models, administration of GSK2394002 not only inhibited structural disease progression but also significantly reduced pain-related behaviors.^47^

M6495 represents an innovative bispecific nanobody therapeutic with dual molecular targeting capabilities. This 28.1 kilodalton (kDa) biologic agent is structurally composed of two engineered variable domains derived from heavy-chain-only llama antibodies.^65^^,^^66^ The therapeutic’s unique design incorporates one domain specifically engineered to neutralize ADAMTS-5 activity, while the second domain facilitates binding to human serum albumin, thereby significantly extending its plasma half-life. Preclinical investigations have demonstrated M6495’s efficacy in inhibiting aggrecan degradation in human cartilage explant models, highlighting its therapeutic potential for OA treatment.^65^ Furthermore, clinical trial data from phase 1/2 studies (NCT03583346 and NCT03224702) have established M6495’s favorable safety profile and tolerability in OA patient populations, supporting its continued development as a promising therapeutic candidate.^67^

## Anti-TNF-α mAbs

OA chondrocytes and synovial cells release TNF-α, a key pro-inflammatory cytokine, which sensitizes pain signaling and enhances the production of pain mediators such as prostaglandin E2 and NGF in synovial fluid and membrane.^68^^,^^69^ *In vitro*, inhibiting TNF-α activity reduces the production of collagenase, matrix metalloproteinases, and other pro-inflammatory mediators in OA cartilage explants.^70^ Three anti-TNF-α mAbs—adalimumab, infliximab, and etanercept—have been tested for OA treatment (see Table 2 for a summary of anti-TNF-α mAbs trials for OA treatment).

**Table 2:** Anti-TNF-α mAbs for OA treatment

Generic name	Identifier (sponsor)	Study phase	Outcome	Main findings	Methods and objectives	Last update	Reference
Adalimumab	NCT00597623 (Hôpitaux de Paris)	phase 3 (completed)	safety, but no efficacy	adalimumab did not outperform a placebo on pain relief in individuals with hand OA who were not responding to analgesics and NSAIDs.	patients received two subcutaneous injections of 40 mg adalimumab at intervals of 15 days, or a placebo, at random, and were followed for six months with hand OA	07-2012	Chevalier et al.^71^
	NCT00686439 (University of Alberta)	phase 2 (completed)	safety and efficacy	adalimumab tends to be useful in lowering symptoms and signs of OA	knee OA patients received subcutaneous injections of adalimumab over 12 weeks in an open-label study without control	06-2012	Maksymowych et al.^72^
Infliximab	NCT01144143 (Herbert Lindsley, MD)	phase 4 (completed)	safety and efficacy	not available	an RCT study of intra-articular infliximab (100 mg), placebo, and methylprednisolone acetate (80 mg) in knee OA patients	02-2018	–
Golimumab	–	preclinical	efficacy	TNF-α was linked to the OA’s increasing hyaline cartilage degradation. Anti-TNF therapy treatment may alleviate chondrocyte damage	use *ex vivo* chondrocytes model of inflammatory arthritis to reveal the anti-TNF therapy mechanism	08-2021	Park et al.^73^

TNF-α, tumor necrosis factor alpha; NSAIDs, non-steroid anti-inflammatory drugs; OA, osteoarthritis; RCT, randomized controlled trial.

Adalimumab, a fully humanized mAb, neutralizes TNF-α by blocking its interaction with TNF receptors.^74^ While primarily used for autoimmune diseases such as rheumatoid arthritis (RA) and psoriasis, its effectiveness in OA is less established.^75^ In individuals with hand OA who were unresponsive to analgesic non-steroidal anti-inflammatory drugs, adalimumab did not outperform a placebo when it came to relieving pain in a phase 3 clinical trial (NCT00597623).^71^ However, a phase 2 study in knee OA patients demonstrated significant improvements in joint swelling and WOMAC scores (*p* < 0.0001), warranting further phase 3 randomized controlled trial (RCT).^72^

A chimeric bivalent monoclonal antibody, infliximab, combines murine variable domains with human IgG1 and κ constant regions.^76^ In a rabbit model, intra-articular injection of infliximab prevented the progression of induced OA by reducing TNF-α and nitric oxide levels in the synovial fluid, thereby mitigating cartilage degradation.^77^ The safety and efficacy of infliximab were further evaluated in a phase 4, unbalanced, randomized, double-blind pilot study (NCT01144143) for the treatment of knee OA. However, with only 16 patients enrolled, the study’s sample size may be too limited to provide robust evidence of its treatment effect, and no data or related publications are currently available. Consequently, larger and more comprehensive clinical trials are needed. Additionally, etanercept—a human recombinant TNF receptor fusion protein that inhibits TNF signaling—is under investigation in an ongoing clinical trial (NCT02722811).^78^ Similarly, golimumab, a human monoclonal antibody targeting TNF, has demonstrated *in vitro* efficacy as an efficient regulator of inflammatory markers and bone metabolism, markedly reducing levels of CRP, IL-6, and VEGF and alleviating cartilage matrix destruction in chondrocytes.^73^^,^^79^ However, no clinical trials have been initiated with golimumab for OA treatment so far.

## Anti-NGF mAbs

OA patients primarily seek medical attention for pain management, a critical factor influencing disability progression and joint replacement decisions. However, current pain management strategies are limited by the absence of medications that effectively alleviate pain without adverse effects.^79^ NGF is a soluble protein that interacts with two different receptors on the surface of cells: the high-affinity NGF-specific tyrosine kinase receptor (TrkA) and 75 kDa neurotrophin receptor (p75NTR).^80^ NGF plays a crucial role in both pain signaling and neuronal survival. Its expression is upregulated by inflammation and injury, and elevated NGF levels have been linked to chronic pain disorders.^81^ In OA patients, increased NGF concentrations in the joints suggest that anti-NGF therapy could offer an innovative approach to reducing persistent OA pain.^82^^,^^83^^,^^84^ NGF-inhibitor mAbs, such as tanezumab, fasinumab, and fulranumab, have shown significant progress in clinical research for the treatment of OA by preventing NGF from interacting with its receptors, p75 and TrkA (see Table 3 for a summary of anti-NGF mAbs trial for OA treatment).

**Table 3:** Anti-NGF and anti-VEGF mAbs for OA treatment

Generic name	Identifier (sponsor)	Mechanisms	Study phase	Outcome	Main findings	Methods and objectives	Last update	Reference
Tanezumab	NCT00733902 (Pfizer)	anti-NGF	phase 3 (terminated)	efficacy, but not safety	joint damage or rapidly progressing osteoarthritis	intravenous tanezumab, tanezumab in combination with an oral NSAID, an active comparator, or a placebo were administered to knee OA patients	03-2021	Hochberg et al.^85^
	NCT00809783 (Pfizer)		phase 3 (terminated)				06-2012	
Fulranumab	NCT02289716 (Janssen Research & Development)		phase 3 (terminated)			subcutaneous injection in knee OA patients for 16 weeks	03-2019	Kelly et al.^86^
Fasinumab	NCT02447276 (Regeneron Pharmaceuticals)		phase 3 (terminated)			patients with moderate-to-severe OA pain in the knee or hip were randomized to receive fasinumab (1, 3, 6, or 9 mg) or a placebo subcutaneously every 4 weeks	11-2022	Dakin et al.^87^
Bevacizumab	–	anti-VEGF	preclinical	efficacy	directly inhibits catabolic processes	evaluate the blocking effects of bevacizumab in articular cartilage from OA patients	01-2023	Nagai et al.^88^

NGF, nerve growth factor; VEGF, vascular endothelial growth factor; NSAID, non-steroid anti-inflammatory drug; OA, osteoarthritis.

Tanezumab, a humanized mAb against NGF,^89^ has been studied in several large-scale, placebo-controlled, randomized clinical trials for the treatment of OA pain in adults. These studies demonstrated that tanezumab significantly reduced pain and improved physical function compared to placebo.^90^ The neurological safety of both intravenous and subcutaneous tanezumab was assessed across nine phase 3 clinical trials involving more than 5,000 OA patients (NCT00733902, NCT00744471, NCT00830063, NCT00863304, NCT00863772, NCT01089725, NCT00985621, NCT02697773, and NCT02709486). Although tanezumab was associated with an increased risk of adverse events related to anti-phospholipid syndrome, such as hypoesthesia and paresthesia, it did not appear to induce widespread peripheral neuropathy.^38^^,^^91^ However, the global clinical research program for tanezumab was ultimately terminated due to a higher incidence of rapidly progressive OA and an increased rate of total joint replacement compared to control groups.^85^

Another anti-NGF mAb, fulranumab, selectively blocks NGF’s biological effects. Janssen Pharmaceutics conducted four phase 3 randomized studies in patients with hip and knee OA (NCT02336685, NCT02336698, NCT02289716, and NCT02301234) to evaluate the safety and efficacy of fulranumab. However, these trials were prematurely terminated due to an internal strategic portfolio decision, not because of any new safety concerns, according to the official report. Consequently, the limited number of participants precluded drawing any definitive conclusions regarding fulranumab’s effectiveness.^86^^,^^92^

Fasinumab (REGH475), another fully humanized mAb, exhibits high binding affinity to NGF with minimal interaction with other neurotrophin family members.^93^ A phase 2b/3 RCT (NCT02447276) assessing the efficacy, tolerability, and joint safety of fasinumab in OA pain demonstrated that all tested doses significantly reduced pain and improved joint function compared to placebo—even in patients who had previously experienced limited benefits from other analgesics.^87^ However, the incidence of treatment-emergent adverse events, including paresthesia, arthralgia, and infections, was higher in the fasinumab groups compared to placebo.

In conclusion, NGF-inhibitor mAbs alleviate pain and inflammation by inhibiting NGF activity. However, the future of these drugs remains highly uncertain due to side effects, particularly in rapid progressive OA. Therefore, a thorough evaluation of their safety and long-term effects is still needed.

## Anti-VEGF mAbs

VEGF/VEGF-A is well recognized for promoting angiogenesis, monocyte chemotaxis, increased vascular permeability, and vasodilation.^94^ Elevated levels of VEGF expression have been observed in the articular cartilage, synovium, synovial fluid, subchondral bone, and serum in advanced stages of OA patients.^95^^,^^96^^,^^97^^,^^98^^,^^99^^,^^100^ Pathological VEGF signaling in the joint, regulated by various mediators, has been linked to cartilage degradation, osteophyte development, subchondral bone cysts, and sclerosis.^101^^,^^102^ Intra-articular antibodies against VEGF could decrease VEGF receptor (VEGFR) in chondrocytes and synovial cells, as well as inhibit angiogenesis and related nerve ingrowth at the joint to impede the advancement of OA and the resulting pain.^103^ Bevacizumab, a commercially available mAb that blocks VEGF, is currently used to treat conditions such as diabetic retinopathy, age-related macular degeneration (AMD), and various tumors. In a rabbit OA model, intra-articular injection of bevacizumab reduced cartilage degradation, osteophyte development, and synovitis^88^ (see Table 3 for a summary of anti-VEGF mAbs trials for OA treatment). Moreover, in articular cartilage explants from OA patients, bevacizumab directly inhibited catabolic processes while enhancing anabolic activity, thereby exerting a protective effect on the cartilage.^104^ However, bevacizumab is associated with a range of potential adverse events, including hypertension, proteinuria, bleeding, impaired wound healing, gastrointestinal perforation, and an increased risk of thromboembolic events (such as blood clots).^105^ These safety concerns need to be carefully considered in any potential use of bevacizumab for OA treatment, and further research still might be needed to evaluate its safety and efficacy in this context.

## Promise of aptamers

Although antibodies have been extensively studied, several challenges remain to be addressed, including high costs, limited efficacy, adverse side effects, and the need for frequent administration. Aptamers, which are synthetic oligonucleotides that bind to specific targets, hold great promise in addressing these challenges. Their unique properties, such as high specificity, low immunogenicity, ease of modification, and cost-effective production,^106^ suggest that they might provide innovative solutions to some of the limitations associated with antibodies, making them a valuable tool in diagnostics, therapeutics, and other biomedical applications.

Through years of development, two Food and Drug Administration (FDA)-approved aptamer drugs have become commercially available so far: avacincaptad pegol and pegaptanib, also called Izervay and Macugen, respectively.^107^ In 2004, the United States FDA granted approval for pegaptanib sodium (Macugen), an RNA aptamer that inhibits VEGF, to be used in the treatment of all forms of neovascular AMD.^108^^,^^109^ In 2023, the FDA-approved avacincaptad pegol (Izervay), an innovative therapeutic agent developed by Iveric Bio, a subsidiary of Astellas Pharma Inc., for the treatment of geographic atrophy secondary to AMD.^107^ It functions as a complement C5 inhibitor.^110^^,^^111^ Currently, numerous promising aptamers have been investigated in clinical trials (see Table 4 for a detailed list of aptamers’ clinical trials), with many still ongoing, targeting various diseases (such as inflammatory conditions, coagulopathies, and tumors).

**Table 4:** Aptamers on clinical trial as therapeutics

Generic name and company	Mechanisms	Identifier (current status)	Condition	Reference
**Inflammation**				

Emapticap pegol (NOX-E36)/(TME Pharma AG)	anti-CCL2	NCT01547897 (phase 2, completed)	type 2 diabetics with albuminuria	Menne et al.^112^
		NCT01372124 (phase 1, completed)	renal impairment	–
		NCT00976729 (phase 1, completed)	healthy volunteers	–
		NCT010852929 (phase 1/2, completed)	patients with type 2 diabetes mellitus	Park et al.^113^
Lexaptepid pegol (NOX-H94)/(TME Pharma AG	anti-human hepcidin	NCT01372137 (phase 1, completed)	healthy volunteers	Boyce et al.^114^
		NCT01522794 (phase 1, completed	human endotoxemia	van Eijk et al.^115^
		NCT01691040 (phase 2, completed)	anemia of chronic disease in patients with cancer	van Eijk et al.^115^
		NCT02079896 (phase 1/2, completed)	ESA-hyporesponsive anemia in dialysis patients	van Eijk et al.^115^

**Oncology**				

Olaptesed pegol (NOX-A12)/(TME Pharma AG	anti-CXCL12	NCT00976378 (phase 1, completed)	healthy volunteers	van Eijk et al.^115^
		NCT01521533 (phase 2, completed)	relapsed multiple myeloma	van Eijk et al.^115^
		NCT01194934 (phase 1, completed)	healthy volunteers	–
		NCT01486797 (phase 2, completed)	relapsed/refractory chronic lymphocytic leukemia	Steurer et al.^116^
		NCT03168139 (phase 1/2, enrollment)	metastatic colorectal and pancreatic cancer	–
		NCT04121455 (phase 1/2, completed)	incompletely resected, newly diagnosed GBM lacking MGMT methylation	Giordano et al.^117^
AS1411(AGRO001)/Antisoma	anti-nucleolin	NCT00512083 (phase 2, completed	acute myeloid leukemia	–
		NCT00740441 (phase 2, unknown)	renal cell carcinoma	Rosenberg et al.^118^
		NCT00881244 (phase 1, completed)	advanced solid tumors	–
		NCT01034410 (phase 2, terminated)	primary refractory or relapsed acute myeloid leukemia	–

**Coagulation**				

REG1 anticoagulation system (RB006 plus RB007)/Regado Biosciences	coagulation factor IXa	NCT00113997 (phase 1, completed)	healthy volunteers	Benedict et al.^119^
		NCT00715455 (phase 2, completed)	percutaneous coronary intervention	Cohen et al.^120^
		NCT00932100 (phase 2, completed)	acute coronary syndromes; Percutaneous coronary intervention	Povsic et al.^121^^,^^122^^,^^123^^,^^124^
		NCT01872572 (phase 1, completed)	healthy volunteers	Vavalle et al.^125^
		NCT01848106 (phase 3, terminated, clinical hold owing to serious allergic reactions	percutaneous coronary intervention	Lincoff et al.^126^
ARC1779/Archemix	anti-A1 domain of von Willebrand factor	NCT00432770 (phase 1, completed)	healthy volunteers	Gilbert et al.^127^
		NCT00507338 (phase 2, terminated)	angioplasty and stenting for heart attack	–
		NCT00632242 (phase 2, completed)	von Willebrand factor-related platelet disorders	Jilma-Stohlawetz et al.^128^
		NCT00742612 (phase 2, terminated, enrollment slower than expected)	carotid endarterectomy	Markus et al.^129^
		NCT00726544 (phase 2, terminated, enrollment slower than expected)	thrombotic microangiopathy	Cataland et al.^130^

CCL2, pro-inflammatory chemokine C-C motif-ligand 2; CXCL12, C-X-C motif chemokine 12; ESA, erythropoiesis-stimulating agent; GBM, glioblastoma; MGMT, O (6)-methylguanine-DNA methyltransferase.

Similar to the non-covalent chemical combination of antibodies and antigens, aptamers specifically bind to target molecules by the cumulative action, hydrogen bond, static interaction, van der Waals forces, water drainage action, and shape matching.^131^ Their targets range from small molecules—such as amino acids, nucleic acids, metal ions, and toxins—to larger entities like enzymes, growth factors, cell adhesion molecules, and even whole cells, bacteria, and viruses.^132^ Several strategies have been developed to enhance aptamer binding affinity.^133^ In particular, hydrophobic modifications, multivalency, and structural optimizations have all proven effective in increasing binding strength.^134^ Meanwhile, specificity can be further improved using i-motif modifications, ethylenediaminetetraacetic acid (EDTA), primer-free libraries, base mutations, and multiple rounds of negative selection.^135^^,^^136^^,^^137^^,^^138^^,^^139^^,^^140^

**Figure 3:**
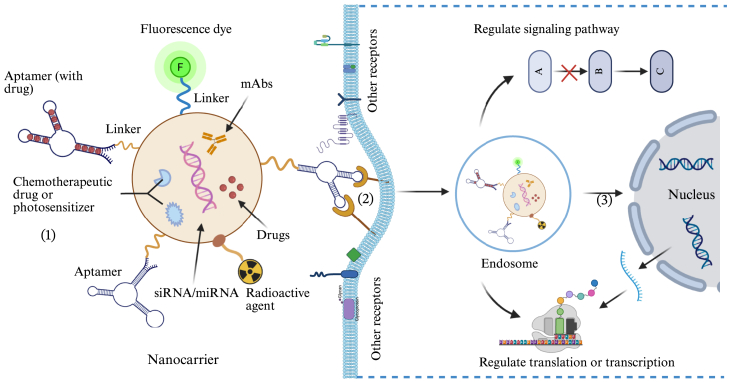
Targeted treatment for OA using functionalized nanocarriers with aptamers specific to cells type To show the target therapy function of aptamer-based nanoparticles for OA, components are assembled in one nanocarrier, including imaging marker (fluorescent dyes or radioactive agents) and therapeutics (mAbs, drugs, oligonucleic acids, and chemotherapeutic agents). mAbs, monoclonal antibodies; siRNA, small interfering RNA; miRNA, microRNA; RNA, ribonucleic acid.

Additionally, aptamers have the appealing feature of being more easily coupled with functional DNA nanostructures than antibodies and tiny peptides.^141^^,^^142^^,^^143^ Due to their simple structures, when compared to more complex structures such as antibodies, aptamers are relatively easy to modify.^144^^,^^145^ They can be modified with precise site-specific adjustments, such as fluorescent, electroactive substances, nanomaterials, biotin, and enzyme labeling. In theory, there would be three potential therapeutic pathways for aptamers: (1) extracellular effects—regulate cell activity by altering the extracellular microenvironment and inflammatory pathways; (2) inflammation pathway—regulate the inflammation signaling pathway by binding to the cell membrane or a receptor-mediated endocytosis route; and (3) regulate the transcription and translation processes of related genes in cells (see Figure 3). Gold nanoparticles (NPs), liposomes, micelles, polymer NPs, mesoporous silica, and dendrimers have all been employed to modify aptamers for use as drug carriers.^20^

## Aptamer studies in rheumatoid arthritis and inflammation

Aptamers may have significant therapeutic potential in the treatment of arthritis, particularly rheumatoid arthritis (RA), through their ability to target key inflammatory pathways and molecules. One notable example is SL1026, an aptamer designed to antagonize IL-6, which demonstrated efficacy in reducing the severity of RA symptoms and postponing the onset of RA in monkeys.^146^ Additionally, single-stranded DNA aptamers with high affinity for connective tissue growth factor (CTGF) have shown promise in RA treatment. Citrullinated aptamer-based NPs decreased autoantibodies and immune responses involved in RA pathogenesis, exhibiting antiproliferative and antiangiogenic effects in a collagen-induced arthritis mouse model.^147^ Another CTGF-specific aptamer, AptW2-1-39-PEG, developed using CTGF protein-based SELEX, not only enhanced diagnostic detection efficiency but also inhibited pannus formation, a key pathological feature of RA.^148^

Aptamers targeting other inflammatory mediators have also shown therapeutic potential. The anti-MUC1/Y aptamer (mucin 1 isoform Y) has demonstrated remarkable efficacy in reducing edema and neutrophil migration in a mouse RA model.^149^ In a study, the anti-interleukin-17A (anti-IL-17A) aptamer effectively blocked the biological effects of IL-17A and suppressed the development of autoimmunity in mouse joint inflammation models.^150^ Furthermore, an aptamer developed in 2022 through the protein-SELEX procedure exhibited potent blocking and neutralizing activity against human IL-17A, achieving over 85% inhibition efficacy in HaCaT cells. This aptamer represents a promising alternative to mAbs as a targeted anti-IL-17A therapeutic agent.^151^ Also, a DNA aptamer inhibiting TNF-α suggests its potential as a non-immunogenic alternative to antibody-based TNF-α inhibitors in C57BL/6 mice.^152^ Additionally, aptamers targeting TNF receptor 1 (TNFR1) were explored as selective inhibitors for RA. These aptamers specifically blocked TNF-α-TNFR1 signaling while leaving TNF-α-TNF receptor 2 interactions unaffected, providing a promising avenue for the development of more targeted anti-RA therapies.^153^ Homodimeric soluble γc (sγc) was evaluated to be a driver of T helper 17 cell differentiations in RA. An sγc-binding DNA aptamer disrupted its dimerization, restored IL-2/IL-15 signaling, and alleviated arthritis in a collagen-induced arthritis model.^154^ Moreover, RNA aptamers targeting the human IL-6 receptor enabled receptor-mediated internalization while maintaining IL-6R signaling in murine cell lines transfected with human IL-6R.^155^ Also, a 2′-fluoro-pyrimidine modified RNA aptamer 8A-35 binding and neutralizing human IL-8 with a high degree of specificity and affinity was identified, which possibly could be used to inhibit neutrophil activity. This could be used as a therapeutic agent for inflammatory diseases, such as OA.^156^

Aptamers developed for RA and other inflammation diseases may also hold significant therapeutic potential for OA, as both conditions share common molecular targets critical to disease development and progression. These targets are deeply rooted in overlapping pathogenic mechanisms and signaling pathways. However, despite this overlap, research on the application of aptamers in OA remains limited, highlighting a need for further exploration in this area.

## Aptamer studies in bone diseases

Research in other bone-related diseases, such as osteoporosis, infections, and cancer, highlights the versatility of aptamers compared to antibodies, which are often limited to specific indications. For instance, CH6 aptamer-functionalized lipid NPs have been used to deliver osteogenic siRNAs directly to osteoblasts, promoting bone formation, improving bone microarchitecture, and enhancing mechanical properties in osteopenic rodents.^157^^,^^158^ Similarly, aptamers targeting receptor activator of nuclear factor kappa-B ligand have shown promise as a therapeutic strategy for osteoporosis.^159^ Given that osteoblasts play a crucial role in OA by contributing to abnormal bone remodeling and subchondral bone sclerosis, regulating bone metabolism through aptamers could offer a novel approach to modifying OA progression.^160^ Despite the differences between bone tumors, osteoporosis, and OA, they share similarities in bone microenvironment regulation and signaling pathways, such as Wnt/β-catenin and TGF-β.^13^^,^^161^ Thus, the cross-disease application to OA might be particularly effective, especially in modulating inflammation, mitigating cartilage degradation, and regulating bone remodeling.

## Aptamer application in OA

The current focus of aptamers for OA has been on targeting cells and blocking inflammatory pathways. For instance, the aptamer RA10-6 effectively blocked IL-17 binding to IL-17RA in a dose-dependent manner in OA mice.^162^ Similarly, RNA aptamers targeting FGF 2 (FGF2), a key player in bone and cartilage remodeling, have shown promise in restoring chondrocyte proliferation and differentiation in preclinical models.^163^^,^^164^ RBM-007, an anti-FGF2 aptamer, is currently under clinical evaluation for achondroplasia and AMD (NCT03633084 and NCT04200248), but its potential application in OA remains unexplored. This is related to OA therapy because FGF2 is implicated in OA pathophysiology.^158^ Additionally, RBM-010, created by Ribomic Inc., was the first RNA aptamer inhibitor specifically designed for ADAMTS-5.^165^ It has been subjected to clinical assessments for the treatment of OA without any results presented so far. Other anti-ADAMTS-5 aptamers, apt21 and apt25, were able to selectively target ADAMTS-5 without binding to other molecules such as blood serum albumin and ADAMTS-4.^166^ Among the ADAMTS family, ADAMTS-5 might be identified as the primary aggrecanase driving OA progression as mentioned previously.^59^ Meanwhile, a CD200R (CD200 receptor) agonist DNA aptamer was developed to modulate inflammation in OA secondary to injury.^167^^,^^168^^,^^169^ However, it is important to note that when using a positive-only SELEX strategy, where counter-selection is omitted, there is an increased efficiency in isolating strong target-binding aptamers, but also a higher risk of identifying candidates with non-specific interactions.

Targeted delivery systems using aptamers may also advance OA treatment. For example, a study investigated the efficacy of delivering microRNAs (miRNAs) to chondrocytes for OA treatment using PEGylated NPs functionalized with FGF receptor 1 (FGFR1)-specific aptamers.^170^ CX3-LS-DQ, an aptamer-functionalized liposome encapsulating senolytics (daratinib and quercetin-DQ), demonstrated a considerable affinity for senescent fibroblast-like synoviocytes. Intra-articular injection of CX3-LS-DQ successfully reduced cartilage breakdown *in vivo* in OA mice model by inducing the apoptosis of senescent fibroblast-like synoviocytes.^171^

Aptamers have also been employed to modulate gene expression in OA. The targeted overexpression of miR-29b in bone marrow-derived mesenchymal stem cells was achieved by delivering aptamer-agomiR-29b. This approach successfully reversed subchondral bone loss and reduced the excessive activity of osteoclasts in OA mice with unilateral anterior cruciate ligament injury.^172^ Furthermore, DEK-targeting (DEK proto-oncogene) DNA aptamers reduced inflammation and neutrophil extracellular trap formation in inflammatory arthritis mice models. Enhanced stability and transdermal delivery of the DEK-targeting aptamer DTA via a hydrogel microneedle system further demonstrated its therapeutic potential in reducing joint damage in a collagen-induced arthritis model.^173^^,^^174^

Although preclinical data on aptamers for OA remain limited, promising results from current studies show significant promise in OA therapy by targeting inflammatory pathways, modulating gene expression, and enabling precise drug delivery. By leveraging the SELEX technique to develop aptamers that specifically target joint cells or inhibit key inflammatory signaling pathways, researchers may overcome existing therapeutic limitations and achieve breakthroughs in managing OA.

## Advantages and limitations of mAbs for treating OA

Aptamers and mAbs are two distinct classes of therapeutic molecules with unique properties and mechanisms of action. They are both types of biologics that can specifically bind to target molecules with high affinity. However, they differ in their molecular structures, production methods, properties, target substance, modification ability, stability, and applications.^175^ To fully comprehend the distinction between aptamers and antibodies, we made a detailed comparison between mAbs and aptamers in terms of the treatment for OA (see Table 5 for a detailed comparison between mAbs and aptamers).

**Table 5:** Comparison between mAbs and aptamers

Categories	Aptamers	mAbs
Basic composition	nucleotide (A, G, T/U, C)	amino acid
Materials	nucleic acid (single-strand DNA or RNA)	protein (polymer peptide)
Molecular weight/size	6–30 kDa (20–100 nt)	150–180 kDa
Affinity	high (K_D_ ranging from 10^−9^ mol/L to 10^−12^ mol/L); multivalent aptamers have the ability to provide greater affinity and extra functionality	high (K_D_ ranging from 10^−3^ mol/L to 10^−9^ mol/L); the affinity between antibody and antigen is determined by the quantity of identical epitopes present on the specific antigen being targeted
Specificity	high; aptamer can identify single-point mutations and conformational isomers	high; antigens may have multiple epitopes, which allow different antibodies to bind to the same antigen
Stability	more stable, easily renatured after denaturation, used repeatedly; resistant to high temperature (even up to 95°C) and cycles of denaturation/renaturation	less stable; susceptible to temperature (even at RT or 37°C) and irreversible denaturation
Potential targets	ATP, amino acids, nucleic acids, metal ions, toxins, enzymes, growth factors, cell adhesive molecules, and even entire viruses, bacteria, cells, tissue slices	antigen proteins
Modification	can be easily modified with precise site-specific modifications, such as fluorescent, nanomaterials, biotin, and enzyme labeling with retaining activity	difficult to be modified and easy to denature; limited types and chemical reactions
Generation/discovery	*in vitro* SELEX (2–15 selection rounds); ∼2–8 weeks; economical to be produced	*in vivo* biological system; ∼6 months or longer; complex and expensive to be generated
Immunogenicity	low immunogenic	highly immunogenic and can cause harmful immunological responses in humans; increased immune reaction with repeated dosing
Batch-to-batch variation	none or low	significant
Tissue uptake/penetration	faster	slower
Kidney filtration	faster, short circulation time *in vivo* (∼30 min for unconjugated version)	slower; long circulation time (up to 1 month)
Development/market	the development pathway is less explored; insufficient education and investment; the commercialization has concentrated on diagnostic-based aptamer products	well-developed infrastructure; abundant financial and educational supports; rapid and sustained increase in medicine market share
Nuclease degradation	vulnerable; limited half-life *in vivo* (∼10 min for unmodified version)	resistant and not affected by nucleases *in vivo*

K_D_, dissociation constant; RT, room temperature; SELEX, systematic evolution of ligands by exponential enrichment.

The study of new mAbs for OA treatment is now the focus of a few clinical trials. mAbs often exhibit a longer half-life compared to small molecule drugs, allowing for less frequent dosing.^176^^,^^177^ Most clinical trials have administered mAbs via intraarticular or subcutaneous injections at 2- to 4-week intervals over periods of 12–16 weeks, which helps enhance patient compliance and convenience while reducing treatment burdens. Although mAbs offer several advantages for targeted OA therapy, many challenges remain unresolved. To date, ongoing trials have not produced satisfying or promising results. Despite decades of clinical investigation, no mAbs have gained approval for OA treatment due to safety concerns and limited efficacy. In particular, mAbs that specifically target individual pro-inflammatory mediators, such as TNF or IL-1β, have largely failed to alleviate OA symptoms. While inhibiting multiple mediators simultaneously may be a more effective strategy, the long-term use of broad-spectrum anti-inflammatory agents carries its own risks. Overall, the development of mAbs for OA appears to have reached significant barriers that may be difficult to overcome in the near future.

## Advantages and limitations of aptamers for treating OA

As mentioned previously, aptamers hold significant theoretical advantages for treating OA, as suggested by their therapeutic potential across several diseases. Many aptamers developed for other inflammatory or degenerative conditions have demonstrated the ability to target common molecular pathways involved in tissue degradation and inflammation. This cross-disease efficacy suggests that aptamers effective in modulating key mediators—such as TNF-α or various interleukins—in diseases like RA or even in oncology could be repurposed or fine-tuned to address the multifaceted pathology of OA. Moreover, the inherent versatility of aptamers enables researchers to adapt these molecules to optimize their binding specificity and stability in the joint microenvironment.

However, there are some limitations that should be taken into account. Unmodified aptamers can be susceptible to nuclease degradation and rapid clearance from the bloodstream, leading to a short half-life *in vivo*.^178^ This may result in a high frequency of repeated administration and lower OA patients’ compliance. Although aptamers are generally less immunogenic than antibodies, they can still elicit immune responses in some individuals, particularly if used repeatedly or in high doses.^179^ The advancement of aptamer technology often faces obstacles due to difficulties in achieving optimal binding affinity and specificity, as well as the intricate processes involved in their modification, evaluation, and refinement.^180^ Additionally, experimental conditions, such as temperature, buffer composition (including ion concentration, ionic strength, and pH), and other variables, can profoundly influence aptamer structures and their interactions with target molecules, potentially leading to false-positive outcomes.^181^

The development of aptamers involves a labor-intensive and costly process, including multiple rounds of selection, refinement, and experimental validation. Although devices and artificial intelligence methods capable of automatically performing SELEX screening have been developed, the process of cell-SELEX remains exceedingly complex.^182^^,^^183^ Therapeutic oligonucleotides, such as aptamers, can induce some adverse effects that require careful monitoring. These include inhibition of blood clotting, activation of the complement cascade, immune system stimulation, and accumulation in tissues.^184^^,^^185^ The latter is often due to the high binding affinity of oligonucleotides for specific tissues, which can hinder their elimination through the kidneys or liver.^186^ However, these effects are generally reversible and considered manageable upon cessation of therapy.^185^

Currently, research on aptamer-antibody complex therapy remains very limited, and no studies have yet explored this approach in bone and cartilage diseases, including OA. Future efforts could focus on the synergistic application of aptamer and antibody to enhance therapeutic effect. Although conjugation of Abs and aptamers has been shown to increase affinity in the case of anti-thrombin antibody-aptamer pincers, this approach has not been explored in OA.^187^ Thus, extensive further research and clinical trials are necessary to validate the safety and effectiveness of this innovative approach.

Overall, while aptamers hold great promise as therapeutic agents for treating OA, addressing these drawbacks and minimizing side effects will be crucial for their successful clinical implementation. Further research and development efforts are still needed to optimize aptamer design, improve pharmacokinetic properties, and overcome translational barriers to bring aptamer-based therapies to patients with OA.

## Conclusion and expectations

Management of OA remains a significant clinical challenge due to its complex pathogenesis and often late detection. While the ongoing development of mAbs offers promising avenues for targeted OA therapy, decades of research and multiple clinical trials have yet to produce an approved mAb treatment, primarily because of limited efficacy and significant side effects. Consequently, alternative approaches are necessary. Aptamers, in particular, hold potential advantages over mAbs, including higher binding affinity and specificity, a broader range of target molecules, easier modifiability, lower immunogenicity, and reduced production costs. Numerous nucleic acid aptamers have been screened, with some demonstrating the ability to alleviate OA symptoms *in vitro* and preclinical models. However, no clinical studies have yet been conducted on aptamer-based OA therapies, and related research is still in its initial stages and needs to be further deepened. Therefore, further investigation into the use of aptamers, along with related modifications and combination treatment strategies, may unlock new and versatile applications for OA therapeutics.

## Acknowledgments

This research was supported by funding from the 10.13039/501100003246NWO Open Competition Domain Science XS (OCENW.XS22.2.148). The PhD studies of Z.Y. (no. 202207720092) and H.C. (no. 202306320055) were funded by the 10.13039/501100004543China Scholarship Council (CSC). The graphical abstract and Figures 1, 2, and 3 were created with Biorender.com.

## Author contributions

All authors were involved in designing the study. H.C. contributed to the conception and design of the review, and was responsible for the literature search, analysis, and drafting of the manuscript. Z.Y. assisted in the literature search and analysis, and contributed to the critical revision of the manuscript. H.W. and L.B.C. provided guidance on the structure and content of the review, and contributed to the critical revision of the manuscript. J.V.K. and J.L.R. provided feedback on the draft manuscript, and contributed to the editing and revision process. J.L.R. supervised the overall project, provided significant input on the design and content of the review, and approved the final version of the manuscript for publication. All authors have read and agreed to the published version of the manuscript.

## Declaration of interests

The authors declare no competing interests.
